# Can monkeys and apes use sequence order to resolve perceptual ambiguity? A lack of evidence in two rhesus macaques and one language trained bonobo

**DOI:** 10.1098/rsos.260293

**Published:** 2026-10-21

**Authors:** Angelle Antoun, Rohini Murugan, Amanda Epping, Jared Taglialatela, Benjamin Wilson

**Affiliations:** ^1^ Department of Psychology, Emory University, Atlanta, GA, USA; ^2^ Emory National Biomedical Research Center, Atlanta, GA, USA; ^3^ Ape Cognition and Conservation Initiative, Des Moines, IA, USA; ^4^ Kennesaw State University, Kennesaw, GA, USA

**Keywords:** language, sequence processing, ambiguity resolution, categorization, visual perception, ape, monkey, bonobo, comparative cognition, evolution

## Abstract

In human language, word order impacts how words are categorized and understood (as nouns, verbs etc.). We have demonstrated that this effect extends to non-linguistic stimuli, suggesting that the impact of sequence order on stimulus categorization is a domain-general, rather than language-specific cognitive process. Here, we ask if this process is evolutionarily conserved in other non-human primates. We developed a non-linguistic experiment and tested rhesus macaques (*Macaca mulatta*) and bonobos (*Pan paniscus*). Participants first learned to categorize shapes (as rounded, squared or pointed). We next assessed how they categorized ambiguous morph shapes by combining exemplars from each category. We provided sequential information by training the animals to select shapes in a specified order: rounded, then squared and then pointed. Finally, we assessed whether presentation in specific positions in this learned sequence would impact subsequent categorization of ambiguous morph shapes. In humans, an ambiguous morph was more likely to be categorized as the shape it replaced in a sequence. Non-human primates, however, showed no such effect, categorizing stimuli independent of their position in a sequence. This suggests that the tendency to infer category membership from sequence order is not shared across primates, and may have evolved more recently in the human lineage.

## 1. Introduction

In language, word order is critical for interpreting the meaning of a sentence. For example, in the sentence ‘the dog chased the cat’, word order dictates the subject (in this case, the dog) and the object (the cat) of the sentence, and specifies who did what to whom. Moreover, grammatical rules are applied to linguistic categories such as nouns and verbs, and word order can also disambiguate to which of these categories a word belongs [1–3]. For example, in the sentences ‘book a place’ versus ‘place a book’, the order of the words clarifies which word is the noun and which is the verb; word order directly impacts how words are perceived, categorized and understood. While this process is central to language, similar processes can be observed in non-linguistic contexts [4] and may even exist in non-human animals. To assess this question and elucidate the evolutionary origins of the interaction of sequence and category information, here we explored whether similar processes might be seen in closely related non-human primates, rhesus macaques (*Macaca mulatta*) and bonobos (*Pan paniscus*).

We recently developed an experimental paradigm to assess the generalizability of the interaction of word order and grammatical category to non-linguistic contexts in humans [4]. This study showed a pronounced impact of sequence order on how ambiguous shape stimuli were categorized. Participants learned to select shape stimuli in a specific order based on their perceptual properties (e.g. rounded → pointed → squared). When we then presented ambiguous stimuli (e.g. a morph shape that was both rounded and pointed), to be selected in a particular order within that sequence (e.g. replacing the rounded shape), it was much more likely to be subsequently categorized as rounded than pointed. The effect of sequence order on categorization, even in a non-linguistic context, suggests this might in fact be a domain general ability rather than a language specific one. This therefore raises question about whether the ability to infer category from sequence may be an older, evolutionarily conserved ability, or if it may be a more recent ability that still relies on language systems, even if it can be applied to non-linguistic stimuli. If this ability predates the evolution of language, it may be part of the suite of cognitive abilities from which language evolved.

Even outside of language, structured patterns in sequences of events are ubiquitous across environments, and the ability to detect regularities in these patterns is essential for perception, action and cognition. Sensitivity to sequential information is thus crucial for the survival of many animals, including primates. For example, chimpanzees are capable of extracting honey from hives within trees using a variety of types of tools, each of which must be used in a specific order to successfully achieve a particular part of the extractive foraging process [5]. During communication, many species systematically produce vocalizations or gestures in specific orders [6,7]. Monkeys are even sensitive to the order in which the vocalizations of conspecifics appear in a sequence (e.g. [8]), and some can group those vocalizations into familial categories and organize those categories into sequential hierarchies [9]. Therefore, it is possible that sensitivity to the order in which events, or categories of events, occur might be broadly conserved across primate species.

Historically, non-linguistic experimental paradigms have been used to assess language-relevant cognitive abilities in non-human animals. For example, comparative artificial grammar learning experiments have demonstrated that a range of sequence learning abilities are evolutionarily conserved in multiple species [10–13]. This raises the possibility that language, complex as it is, may be built on evolutionarily older cognitive systems that can be assessed across species [14,15]. Determining the presence of other language related abilities in non-human primates can help map the evolutionary trajectory of these component abilities, clarifying how and when various aspects of the human language system may have evolved. Given that non-human primates are sensitive to the order of events in the environment in a range of contexts [5,7–9], it is possible that they might also use sequence order to resolve category ambiguity. If so, this ability may represent a precursor to its current linguistic role in humans.

Here, we adapted our previous experiment for use with non-human primates, to assess whether they use sequential information to resolve ambiguity, as humans do in language. Doing so could elucidate evolutionarily conserved cognitive abilities onto which language may have been built. We use a similar method in both rhesus macaques (*M. mulatta*) and bonobos (*P. paniscus*). The direct comparison of humans, to non-human great apes, to monkeys will allow us to assess when this ability may have arisen in the primate lineage. Moreover, one of the bonobos tested, Kanzi, has had significant language training, having learned an extensive vocabulary of lexigrams for communication with human handlers [16]. This allows us to directly compare several potential hypotheses. First, if the tendency to use sequential information to infer category membership is an evolutionarily ancient cognitive process which was later co-opted for language, we would expect both macaques and bonobos to show a strong effect of sequence on categorization. If, on the other hand, neither non-human primate species shows this effect, it would suggest it may be a human-unique specialization, emerging either as a direct result of the evolution of language, or based on other recently evolved cognitive processes which are unique to humans. It is also possible we will find this effect in bonobos, but not macaques. This would suggest a more recent specialization in the great ape lineage. Finally, an effect in Kanzi alone, but not in the other bonobos or the macaques, could suggest this effect is the result of explicit training and experience with language.

## 2. Methods

### 2.1. Participants

Two rhesus macaques (female, ages 4–5 years) and four bonobos (three males, ages 11–41 years and one female, age 14) began this experiment. However, owing to the length and difficulty of the task, only one bonobo completed the initial training and progressed to the key testing phase. Macaques were housed at the Emory National Biomedical Research Center, Atlanta, GA, USA. The room was on a 12 L : 12 D cycle, with lights on between 8.00 and 20.00. They were typically pair housed but were separated from each other by a plastic divider for testing. They were given free access to water throughout the day, and, in addition to food rewards received for correctly completing trials, daily calories were provided at the end of the day in the form of biscuits (LabDiet) and produce. We tested bonobos at the Ape Initiative, IA, USA. Bonobos were housed in fission–fusion mixed-sex social groups typically including three to four individuals. Bonobos were given free access to water and food (fresh fruits and vegetables, nuts and seeds etc.) throughout the day. Individual subjects were asked to voluntarily separate from the group for testing. All ethics were approved by Emory University IACUC (PROTO202100030) and The Ape Cognition and Conservation Initiative IACUC 210303-01.

### 2.2. Stimuli

The goal of this experiment was to assess the role of sequence order on shape categorization in non-human primates (based on methods developed in Antoun & Wilson [4]). We generated three perceptually distinct categories of stimuli: ‘rounded’ (A), ‘squared’ (B) and ‘pointed’ (C) shapes (figure 1a). Shapes had between 4 and 10 limbs protruding from their centre, and there were three variations in the width of the limbs, resulting in 21 shapes per category for 63 total shape stimuli (figure 1a). All stimuli were white shapes on a black background (200 × 200 pixels) and were generated using custom code in MATLAB R2021b (see the supplementary material).

**Figure 1.**
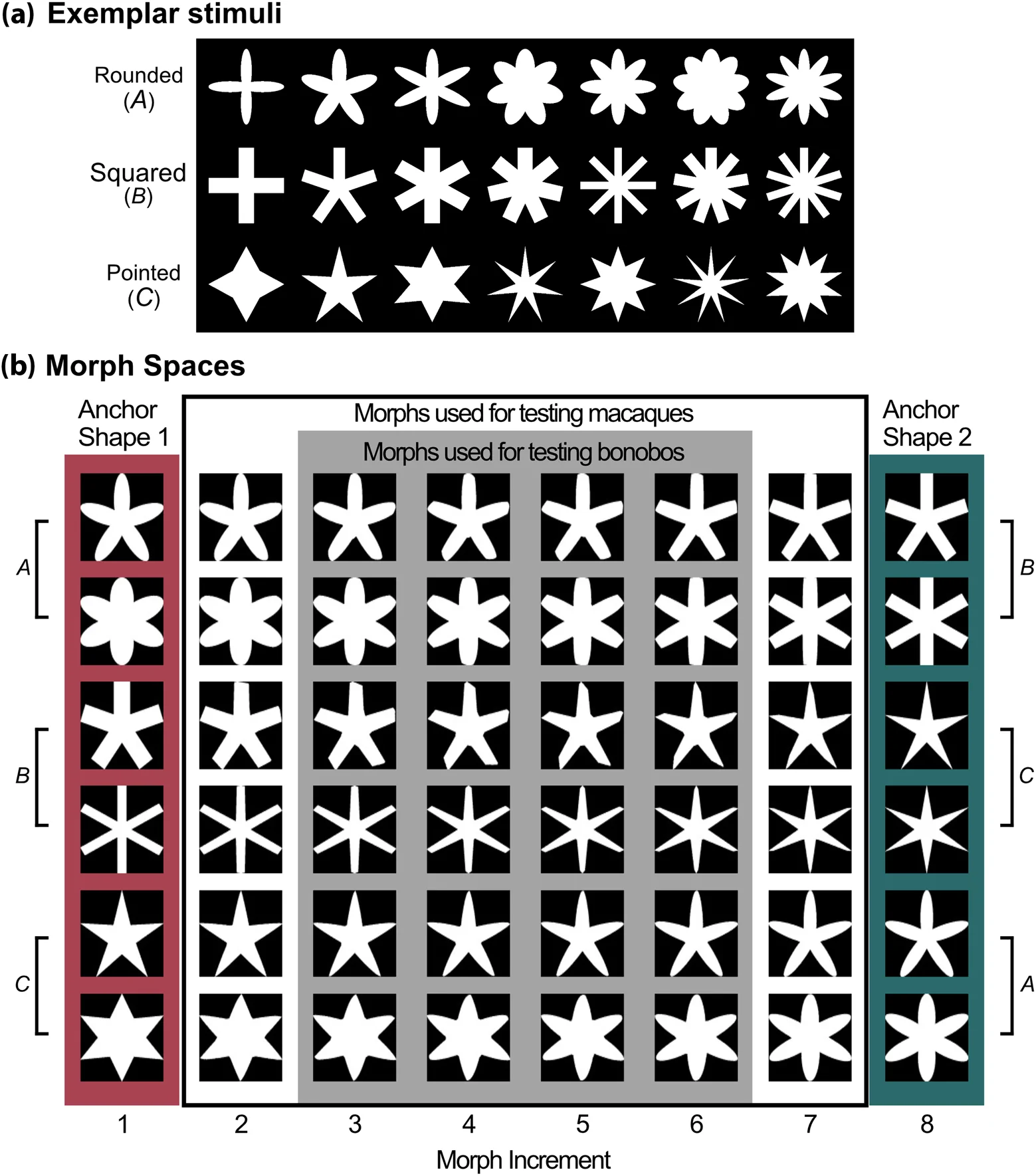
Stimuli. (a) Exemplars from each stimulus category. Rounded (A), squared (B) and pointed (C) shape stimuli. Each category included 21 items, ranging from 4 to 10 limbs, each with three variations of width and length (a random subset of which are shown here). (b) The six morph spaces. Each of the anchor 1 shapes (in the left column, highlighted in pink) was incrementally morphed with the anchor 2 shape in the same row (right column, highlighted in blue), generating six intermediate morph ‘increments’ that resemble combinations of the two, ranging from one to the other. Only the four most ambiguous increments (3, 4, 5 and 6, highlighted by the grey background) of each morph space were used as test stimuli for bonobos, with increments 2 and 7 added for macaques (white box).

For categorization tests, ambiguous morph shapes were generated by selecting shapes from two different categories to act as ‘anchors’ (e.g. a rounded (A) and a squared (B) shape), and interpolating between points around their circumference to generate a morph space (e.g. AB; figure 1b). We created a total of six morph spaces: one exemplar with five limbs and one with six limbs for each of the three combinations of categories (AB morphs, BC morphs and CA morphs; figure 1b). From each of these six morph spaces, six intermediate morph shapes were generated at equal increments between the two anchors (figure 1b). For the macaques, all six of these ambiguous morphs were used. For the bonobos, who performed significantly fewer trials each day, only the four most ambiguous morph shapes from each morph space were used for testing to allow for more data collection at the points of greatest ambiguity. This resulted in a total of 36 morph shape stimuli for the macaques and 24 for the bonobos.

### 2.3. Apparatus

All participants were temporarily separated from their groupmates for testing. Macaques were trained and tested using custom cognitive testing systems, comprised of a touchscreen monitor (ELO) controlled by a laptop computer (Dell), and a pellet dispenser (Med Associates) that provided food rewards (TestDiet primate pellets). These were attached to the door of each animals' enclosure for approximately 6 h d^−1^, and the animals were freely allowed to interact with the screen. Bonobos were tested with a similar system, consisting of a touchscreen monitor (ELO) and laptop computer (HP), but were manually handed food rewards (pieces of fruit or other preferred food items) by a keeper for correctly completing trials. Bonobos were tested for much shorter periods of time each day (typically less than 15 min d^−1^), therefore fewer trials were possible in this species (see below, and table 1).

**Table 1.** Final performance on each phase of the experiment. (For the training phases (match to sample, phase 1 and phase 3), performance is reported over the last five sessions. For phases 2 and 4 (which were the key testing phases) data are included from the last five sessions as well, though there was no performance criterion for those phases. The trial was considered correct if either anchor shape of the morph was selected, and incorrect only if the third unrelated shape was selected. We also report the duration of each phase in both trials and sessions.)

	match to sample	phase 1	phase 2	phase 3	phase 3.5	phase 4
**macaques**						
**Jasper**	**75%** (19 793 trials; 11 sessions)	**76%** (28 685 trials; 19 sessions)	**79%** (12 710 trials; 10 sessions)	**56%** (6433 trials; 6 sessions)	**seq: 62% cat: 74%** (4753 trials; 5 sessions)	**seq: 79% cat: 85%**(11 372 trials; 10 sessions)
**Lapis**	**72%** (19 101 trials; 19 sessions)	**70%** (34 448 trials; 53 sessions)	**75%** (13 696 trials; 15 sessions)	**63%** (4791 trials; 8 sessions)	**seq: 90% cat: 71%** (8589 trials; 12 sessions)	**seq: 80% cat: 82%** (10 596 trials; 17 sessions)
**bonobos**						
**Kanzi**	**73%** (2660 trials; 50 sessions)	**71%** (5935 trials; 95 sessions)	**83%** (1056 trials; 22 sessions)	**66%** (4570 trials; 137 sessions)	**seq: 63% cat: 70%** (166 trials; 5 sessions)	**seq: 58% cat: 83%** (2176 trials; 23 sessions)
**Teco**	**94%** (11 901 trials; 236 sessions)	**56%** (3891 trials; 62 sessions)				
**Nyota**	**57%** (6888 trials; 136 sessions)					
**Mali**	**54%** (2730 trials; 65 sessions)					

### 2.4. Procedure

The experiment consisted of four phases. In all phases, stimuli were presented on a touchscreen monitor, and participants touched them to make responses (see below, and figure 2). Feedback was given in the form of a green or red screen, and a positive (‘ding’) or negative (‘buzz’) sound played through audio speakers. Correct responses resulted in a food reward (macaques received a primate pellet, bonobos received a small piece of fruit), and incorrect responses resulted in a brief ‘time-out’ that delayed the start of the next trial. Prior to the beginning of this task, all individuals were trained on a match to sample task (in which they were presented with a shape cue and were rewarded for selecting the matching shape from an array of possible targets) to familiarize them with the experimental paradigm. Unfortunately, two of the bonobos failed to complete this training, therefore only two advanced to the main experiment (table 1).

**Figure 2.**
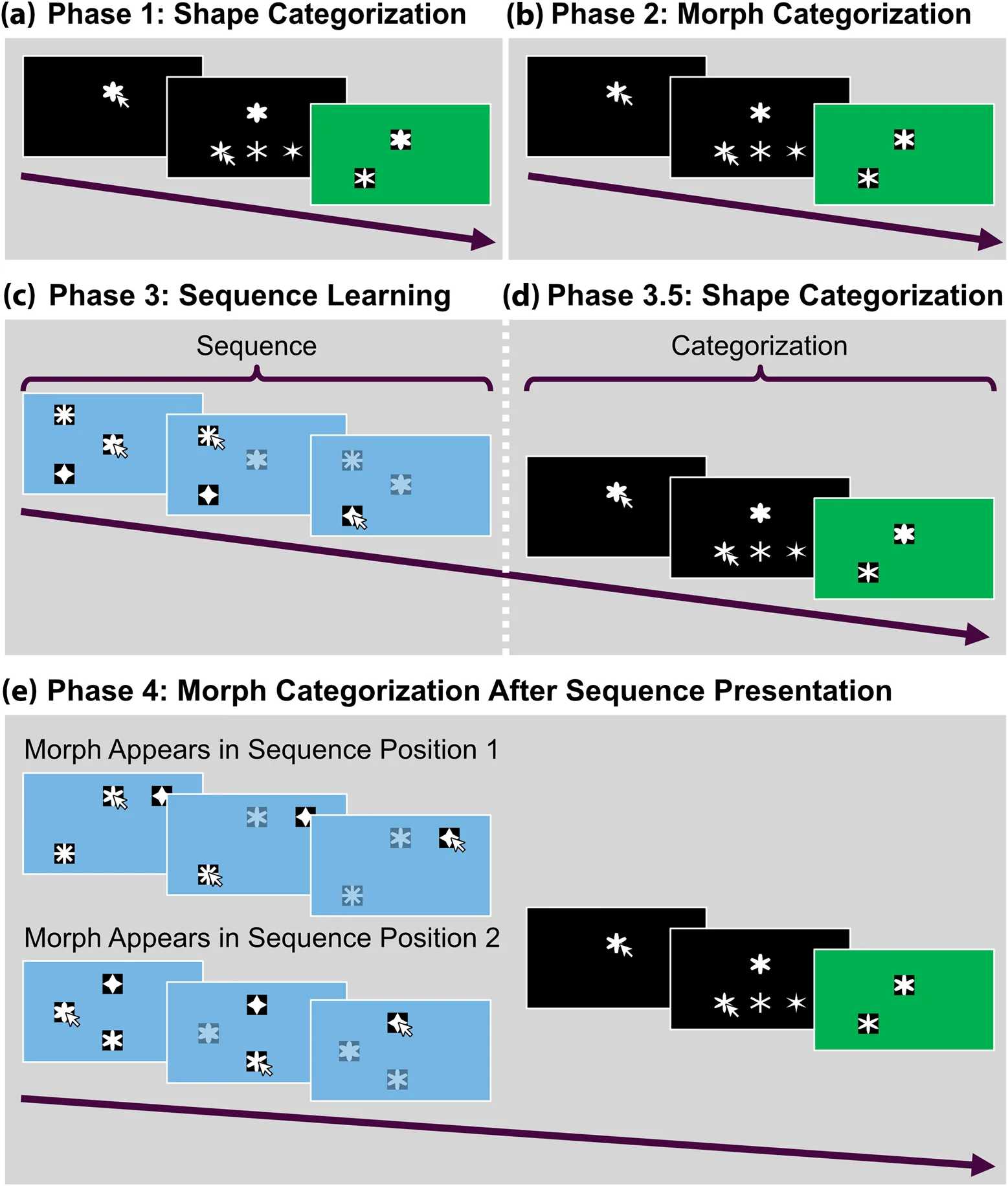
(a) Phase 1: an example shape categorization trial. Participants were presented with a cue stimulus, and then selected the target stimulus from the same shape category. (b) Phase 2: an example morph categorization trial. An ambiguous morph was presented as a cue stimulus, and participants selected the shape category that they determined most closely resembled the cue. (c) Phase 3: an example of a sequence learning trial. Participants were presented with shapes from the three different categories and learned to select them in a specific sequence order (rounded (A) → squared (B) → pointed (C)). (d) Phase 3.5: an example of adding shape categorization to a sequence trial. Subsequently, a shape categorization portion (identical to phase 1) was added to the ends of the sequence learning trials, using one of the three stimuli from the sequence as the cue stimulus. (e) Phase 4: an example of a sequence presentation and morph categorization trial. Participants were presented with an ambiguous shape (e.g. AB) alongside two standard stimuli (either B and C (upper panel), or A and C (lower panel)). They were then required to attempt to select these in order based on the A → B → C sequence, with the ambiguous morph taking the place of the missing shape category within the sequence. In the first example, the morph (AB) replaces the first anchor shape (rounded, A), so participants should select, AB → B → C. In the second example, the morph replaces the other anchor shape (squared, B), so participants should select, A → AB → C. Immediately following this sequencing trial, participants were then given a final shape categorization trial in which the ambiguous morph (AB, in this case) was used as the cue stimulus.

### 2.5. Phase 1: initial category training

Participants first learned to categorize the shape stimuli. On each trial, they were presented with a randomly selected shape (hereafter the ‘cue stimulus’) in the centre of the screen. After touching the cue stimulus, an exemplar shape from each category (rounded (A), squared (B) and pointed (C); hereafter the choice stimuli) appeared at the bottom of the screen in three randomized positions. The choice stimuli were randomly selected from shapes with the same number of limbs as the cue, but different width parameters such that the correct choice was not identical to the cue (figure 2a). Participants were given positive feedback if they selected the choice shape of the matching category, and negative feedback for any other selection. Testing continued until they reached our specified criterion (70% performance overall that day, where chance performance on each trial was 33.3%) for two out of three consecutive days. Despite appearing to be a relatively simple task, learning was slow, taking many thousands of trials, and of the two bonobos who began this phase of the experiment, only one completed it (table 1). Therefore, we present only data from the single bonobo, Kanzi, who advanced to the testing phase.

### 2.6. Phase 2: baseline categorization

Next, we assessed how each participant categorized the ambiguous morph shapes. The procedure for phase 1 was repeated, but now ambiguous morph stimuli were presented as cue stimuli on some trials (see below, figure 2b). In these probe trials, the choice stimuli were the two anchors from which the morph shape was generated, and a stimulus from the third category with the same number of limbs. In macaques, ambiguous probe trials made up 4 out of each 11 trials presented (each testing block consisted of all 63 standard stimuli intermixed with all 36 morph shapes). Macaques participated in a minimum of 50 blocks in this phase, continuing until they had categorized each morph shape at least 50 times. Bonobos were willing to work for fewer trials in a day than macaques, so for bonobos, we presented the ambiguous morph stimuli on 50% of trials (each session included 24 randomly selected standard trials, alongside the reduced set of 24 morph shapes used with the bonobos). The one bonobo who reached this stage completed 22 blocks in this phase.

### 2.7. Phase 3: sequence training

Participants were next taught to select shape stimuli in a specific order: (rounded (A) → squared (B) → pointed (C); figure 2c). Participants were presented with a randomly selected set of three shapes, one from each category, randomly positioned on the screen. Initially, these shapes were presented in sequential order; however, this made it possible to rely on presentation order rather than shape identity. We therefore subsequently presented the shapes simultaneously and participants were required to select them in the correct A → B → C order. During this sequencing stage, the screen background was recoloured blue, to provide a cue to the animals that this was the sequencing phase of the task. If they selected any shape in the incorrect order, they received negative feedback, and the same trial was repeated. If they selected the stimuli in the appropriate A → B → C order, they received positive feedback. Training continued until they reached our learning criterion (70% performance overall for at least two out of three consecutive days. Chance performance on each trial was 16.7%). All participants who reached this phase completed it.

Only data from the simultaneous presentation version are reported here. However, various interventions were attempted to aid Kanzi in learning the sequence. First, we tried using sequential presentation, and using motion cues. Stimuli appeared in order separated by a 1 second inter-stimulus-interval, followed by a brief bouncing animation for the stimulus that was meant to be selected. However, learning in this condition did not transfer to the stationary sequence. Next, we added a start signal to encourage the participant to attend to the screen from the start of the trial, to direct attention to the stimulus presentation. We also began repeating trials in which he made errors, rather than moving on to the next trial, to ensure that successful trial completion was required to advance. These interventions did not substantially improve Kanzi's performance. Finally, we added an intervention where the three items appeared in order, but initially presented as transparent and un-selectable. Only once all three stimuli had appeared would they become opaque and selectable. This intervention was successful in improving Kanzi's learning, and when it was removed and the items once more appeared simultaneously as opaque shapes, learning transferred.

After learning to correctly sequence the shapes, we then added a categorization stage to the end of each trial (figure 2d). This part of the trial was identical to phase 1, with a black background to cue the animals that they were in the categorization phase of the task. This categorization stage featured one randomly selected shape previously presented in sequence as the cue stimulus. To correctly complete these trials, participants were required to first select the stimuli in order and then correctly categorize the shape. They continued this training until reaching criterion (over 70% on both the sequencing and categorization tasks for at least two out of three consecutive sessions for the macaques, and 65% for the bonobo. Chance performance on each trial was 5.56%).

### 2.8. Phase 4: testing the effect of sequence position on categorization

The final testing phase examined whether the categorization of ambiguous morph stimuli changed based on the position in which they appeared in the sequence earlier in the trial. On each trial, participants were first required to select the three shapes in the A → B → C sequence, followed by a categorization test (figure 2e). In this phase, intermixed with standard trials (identical to those presented in phase 3), we included morph ‘probe’ trials. In these probe trials, we presented an ambiguous stimulus (e.g. BC) alongside two standard stimuli (e.g. rounded (A) and squared (B)). Participants were required to try and select the stimuli in order, (following the previously learned A → B → C sequence), but the presence of the ambiguous morph required that they now instead selected A → B → BC, with the BC morph replacing the missing C stimulus. Each morph shape was presented in place of each of the two anchor shapes that was used to generate it equally frequently. For example, in half the trials, a squared–pointed morph (BC) would appear in place of the squared (B) shape in the sequence (and thus should be selected second, A → BC → C), and in the other half of trials, it would replace the pointed (C) shape in the sequence (and thus should be selected last, A → B → BC). Selecting stimuli in the incorrect order aborted the trial early, and resulted in negative feedback for the participant. In the second part of the trial (the categorization test, similar to phase 2), the ambiguous morph from the sequence (BC in this example) was presented as the cue stimulus. As in phase 2, the choice stimuli were the two anchors from which the morph shape was generated, and a stimulus from the third category with the same number of limbs. Selection of the third unrelated shape was considered incorrect, and resulted in negative feedback for the participant. These trials were not included in subsequent analyses. For the macaques, we intermixed probe trials with standard trials at a ratio of 72 : 63 (in each batch, all standard stimuli were presented once for categorization, and all morph stimuli were presented twice for categorization, once after replacing anchor shape 1 and once after replacing anchor shape 2). Macaques completed a minimum of 50 such batches of testing, continuing until they had categorized each morph shape in each position a minimum of 50 times. For the bonobo, who completed far fewer trials, we omitted the standard trials and presented only morph trials (48 per batch) to allow us to complete data collection. We collected 22 batches of stimuli in the bonobo.

### 2.9. Data analysis

Following the methods of Antoun & Wilson [4], we first calculated a baseline categorization function for the ambiguous morphs in phase 2. For all ambiguous morph stimuli (six morph spaces with four increments each for bonobos and six increments for macaques, resulting in 24 or 36 total morph stimuli, respectively; figure 1), we measured how frequently each morph (e.g. AB) was categorized as anchor shape 1 (A in this case) versus anchor shape 2 (B in this case). For a CA morph, C was coded as anchor shape 1 and A as anchor shape 2, and for a BC morph, B was anchor shape 1 and C anchor shape 2, to allow us to compare the different types of morphs directly relative to their respective anchor shapes. From this, we generated a baseline categorization curve for each individual aggregated across morph type, by plotting the proportion of trials on which the individual chose anchor shape 2 across all morphs for each given increment (solid purple lines in figure 3). We repeated this process for the morph probes in phase 4, but this time separated the data based on the position in which the morph shape appeared in the preceding sequence. For example, if an AB morph replaced the A shape in the sequence (its anchor shape 1), the categorization of that trial would be included in the shape 1 position condition, whereas if it replaced the B shape (anchor shape 2), it would be included in the shape 2 position condition. This generated two new curves (dotted pink and dashed blue lines in figure 3). Only trials in which participants correctly completed the sequence were included in these categorization data; trials in which the sequence was ordered incorrectly were aborted before reaching the categorization stage. This is because, since we are testing for the impact of position in the sequence, the morph needed to be presented to the participant in that sequence position. Trials in which participants selected the third unrelated shape were also omitted from category analysis as incorrect trials.

**Figure 3.**
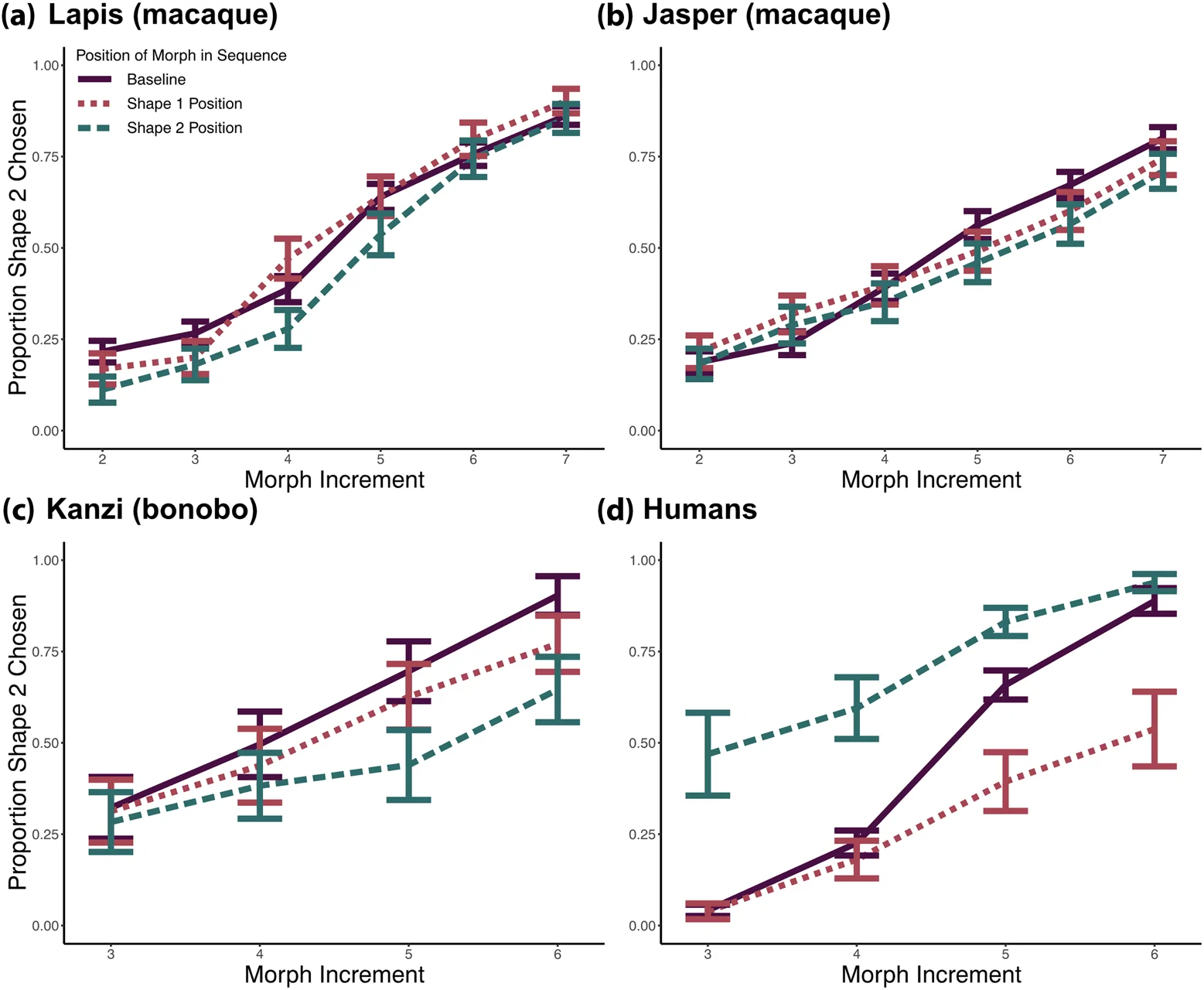
Macaques (*n* = 2) and bonobo (*n* = 1) did not show an impact of sequence on the categorization of ambiguous stimuli. We first assessed how two macaques (Lapis (a) and Jasper (b)) and one bonobo (Kanzi (c)) categorized ambiguous stimuli (e.g. one increment from the AB morph space) as either one anchor (shape 1) or the other (shape 2) to establish baseline patterns of categorization (solid purple lines). After participants learned a simple sequence (A → B → C), the same ambiguous stimuli were presented such that participants selected them in place of one of the two anchor shapes (e.g. AB would be selected as either AB → B → C (replacing A, dotted pink line) or A → AB → C (replacing B, dashed blue line)). The position in which the stimulus appeared in the sequence did not shift categorization in the expected direction for any of the primates, unlike in humans ((d), data from [4]), where ambiguous morphs that replaced shape 1 in the sequence were more likely to be categorized as shape 1 (dotted pink line shifted down compared to purple baseline), and morphs that replaced shape 2 were more likely to be categorized as shape 2 (dashed blue line shifted up from purple baseline).

Given the very small number of participants available (two macaques and one bonobo), analyses were conducted within subjects. A linear model was conducted for each participant to evaluate the impact of increment and sequence position on anchor shape choice. These analyses were run using the base R lm function [17].

## 3. Results

Both macaques and one bonobo (Kanzi) passed all initial training stages, though this required a large amount of training (table 1). The other three bonobos were unable to progress past the first training phase and were therefore omitted from all analyses (table 1).

If the tendency to use sequential information to resolve perceptual ambiguity is evolutionary conserved, then as in humans [4], monkeys and apes' categorization of morph stimuli would be affected by the position in which that stimulus was selected in a learned sequence. For example, if the morph stimulus BC was selected in the sequence A → BC → C, it should be more likely to be categorized as B, and if it was selected in the sequence A → B → BC, it would be more likely to be categorized as C (see human data in figure 3d). In our analyses, we quantified this as the proportion of trials that a morph shape was categorized as anchor shape 2 (choice, in our analyses, figure 3d). We used this metric for our key comparison between baseline trials and trials where the morph replaced anchor shape 1 (in this case B) versus anchor shape 2 (in this case, C) in the sequence. We refer to this as sequence position in our analyses. In humans, we reanalysed the previously collected data from Antoun & Wilson [4] using the same analysis used for the non-human primates here (effect of morph increment × sequence position on choice). In addition to the expected main effect of increment (*F*_3,588_ = 237.03, *p* < 0.0001, $\eta_{p}^{2}$ = 0.55), there was a substantial effect of sequence position on choice (*F*_2,588_ = 188.09, *p* < 0.0001, $\eta_{p}^{2}$ = 0.39; data from [4]), as well as an interaction between sequence position and increment (*F*_6,588_ = 10.32, *p* < 0.0001, $\eta_{p}^{2}$ = 0.10; dashed blue line shifted upwards and dotted pink line shifted downwards in figure 3d; data from [4]).

However, neither of the macaques nor the bonobo demonstrated this effect. While one macaque and one bonobo showed a small but significant main effect of sequence position (Lapis: *F*_2,924_ = 12.88, *p* < 0.0001, $\eta_{p}^{2}$ = 0.03; Jasper: *F*_2,610_ = 2.93, *p* = 0.05, $\eta_{p}^{2}$ = 0.009 and Kanzi: *F*_2,771_ = 11.02, *p* < 0.0001, $\eta_{p}^{2}$ = 0.03), these effects did not occur in any systematic direction, and in fact were opposite to our predictions and the prior human results (compare figure 3a–c with figure 3d). Moreover, none of the animals showed an interaction of sequence position and increment (Lapis: *F*_10,924_ = 1.43, *p* = 0.16, $\eta_{p}^{2}$ = 0.02; Jasper: *F*_10, 610_ = 1.72, *p* = 0.074, $\eta_{p}^{2}$ = 0.03; Kanzi: *F*_6,771_ = 1.18, *p* = 0.32, $\eta_{p}^{2}$ = 0.009), which was the key human result in Antoun & Wilson [4]. This demonstrates that in these animals, sequence position had no impact on shape categorization; patterns of categorization of the morph stimuli did not differ from the baseline period, regardless of where the stimuli were presented in a sequence (figure 3a–c). These results suggest that while there were some subtle differences in how shapes appearing in different sequence positions were categorized, this effect was minimal and not in any predictable direction, in stark contrast to the results previously obtained in humans (compare panels in figure 3).

We did, however, see expected main effects of increment, demonstrating that participants effectively shifted their categorization from one shape to another as the ambiguous morphs shifted in appearance (Lapis: *F*_5,924_ = 244.90, *p* < 0.0001, $\eta_{p}^{2}$ = 0.57; Jasper: *F*_5,610_ = 117.22, *p* < 0.0001, $\eta_{p}^{2}$ = 0.49; Kanzi: *F*_3,771_ = 50.58, *p* < 0.0001, $\eta_{p}^{2}$ = 0.17). These results show that the animals were successfully performing the task and categorizing the morphs in the expected way based on their perceptual features, this categorization was simply unaffected by sequence position.

These results demonstrate that, unlike in humans, in non-human primates, sequence order did not have any impact on how ambiguous stimuli were categorized. This suggests that this process does not appear to be broadly evolutionarily conserved, and might be unique to humans or to language.

## 4. Discussion

Despite evidence that, even in a non-linguistic task, humans' categorization of ambiguous shapes is substantially impacted by the position in which they appeared in a sequence [4], here, two species of non-human primates failed to show this effect.

One of the clearest examples of the importance of sequence order is human language. However, humans are also highly sensitive to sequence order even in non-linguistic contexts [18–20]. Sensitivity to the order in which events occur in sequences is salient and probably crucial for survival for many organisms, for example, in sequences of tool use [5], social interactions [8,9] and communication [7,21]. Therefore, whether this sensitivity arises from language proficiency, or instead represents a more domain-general cognitive process that has been co-opted for language, remains an open question. Comparative research aims to try and address such questions. If we had found similar effects in monkeys, apes and humans, that would suggest an evolutionary origin predating the speciation of these animals (at least 25 Ma [22]). By contrast, evidence that this ability exists uniquely in humans may suggest more recent evolution. Our results support this second conclusion.

Critically, the lack of a sequence categorization effect found here does not arise from lack of ability to learn the task. Both macaques and one bonobo did learn to appropriately categorize stimuli based on their shapes, and to place those shape categories in an ordered sequence. Baseline patterns of categorization for the morph stimuli were similar to patterns found in humans. Morph stimuli that more closely resembled one anchor shape were more likely to be categorized as that anchor shape, while categorization of the more ambiguous morphs vacillated between the two anchors. This indicates that the non-human primates were indeed treating the morph stimuli as ambiguous in their categorization. However, despite learning to complete the task, unlike in humans, sequence order information had no impact on how they then categorized ambiguous stimuli.

Macaques, whose divergence from the human lineage was more distant, approximately 25 Ma [22], as well as bonobos, one of two primate species who share the most recent common ancestor with humans (about 6 Ma [23]), both failed to use sequence order to inform category membership. Given the consistency of the results between the monkeys and bonobo that we tested, this suggests that the tendency to make use of sequential information in categorizing ambiguous stimuli is more recent, and potentially human unique. It is likely that there were differences in how humans and non-human primates encoded the categorical information of the ambiguous morph shapes. Non-human primates, for example, might have been categorizing shapes purely visually, using perceptual information to make each decision. Humans, on the other hand, may have used a different strategy.

Note, these data (and this experiment) cannot differentiate between two possibilities regarding the evolution of this process in humans. Firstly, it is possible that this task engages domain-general cognitive processes separate from those used in language, but that still evolved relatively recently, since our last common ancestor with great apes. Secondly, it is possible that the human experiment recruited participants' language systems, and that they recoded the stimuli linguistically, and processed them somewhat similarly to sentences, despite the use of non-linguistic stimuli. If the latter possibility is the case, ambiguous morphs may have been labelled explicitly during the sequencing phase (e.g. ‘touch round then cross then star’), such that once participants were asked to categorize the morphs, they had already been assigned that specific category. Assessing the explicit use of language during this task would be a valuable next step to clarify the mechanisms at play during this task. Future studies in humans, for example, using linguistic interference tasks, may be required to adjudicate between these two possibilities.

It is also worth noting that the specific bonobo tested in this study, Kanzi, has had substantial language training, and is one of the most proficient non-human primate lexigram users [24,25]. As an individual who has demonstrated potential language-like abilities, a null result from him is particularly strong evidence that bonobos do not exhibit the effect of using sequence information to categorize ambiguous stimuli. This lends support to the conclusion that the tendency to use sequence information to resolve category ambiguity is human specific, and thus evolved relatively recently, after the divergence of the human lineage from other primates, potentially in support of language.

Despite this language experience, our data show no evidence of Kanzi, or either macaque, showing a pattern of results similar to human participants (in [4]). The key interaction of sequence position and increment was not present in any animal, consistent with the conclusion that non-human primates do not use sequence order to aid in ambiguity resolution. Indeed, Kanzi, and both macaques showed a pattern of results opposite to that found in humans (blue lines appear below pink lines in figure 3). In two animals, Lapis and Kanzi, this effect reached significance (small main effect of sequence position) in the opposite direction to the one observed in humans, further demonstrating that the ambiguity resolution effect we demonstrated in humans is absent in these non-human primates. Rather, this result may suggest the animals employed a different strategy to the human participants. During the categorization phase, Lapis and Kanzi were more likely to categorize ambiguous morph shapes as a shape that was present during the preceding sequence (e.g. if presented with the sequence AB → B → C, they were more likely to categorize AB as B, rather than A, in stark contrast to human participants). This might imply that they responded based on which shapes they saw most recently, rather than where they placed the morph within the sequence. Owing to the time and effort involved in collecting this type of data, we only tested three animals (one bonobo and two macaques). Clearly, it is not possible to draw definitive conclusions about all apes and monkeys, or even all bonobos and macaques, from such a small sample size. We therefore encourage the reader to interpret these results cautiously, and certainly welcome the collection of additional data in a larger number of individuals or species.

While neither primate species showed an impact of sequential information on categorization in this task, the interaction of sequence and category may still be relevant and useful to non-human animals [5,7–9,21]. As such, it is possible this task fails to capture this ability. This could be the result of constraints on memory, attention or motivation. If motivation to remember the morph shapes truly is the critical element, this could indicate humans are more intrinsically motivated to ‘solve’ the task, and thus made more explicit attempts to hold the sequential information in mind for later use.

Altogether, we can conclude from these data that while humans spontaneously and automatically make use of sequential information when making categorical decisions, non-human primates do not. Critically, we found no difference in performance between macaques and great apes, implying similar cognitive systems were probably at play across species. This is true even considering that the bonobo in question has significant language experience, so prior use of sequences of symbols is not sufficient to elicit this effect. This points to differences in underlying cognitive mechanisms between humans and other primates, possibly modulated by language, or language relevant systems of rule and pattern searching.

## Data Availability

Data and stimulus creation and analysis scripts are available here [26].
